# Measurements of patient’s setup variation in intensity-modulated radiation therapy of head and neck cancer using electronic portal imaging device

**DOI:** 10.2349/biij.3.1.e30

**Published:** 2007-01-01

**Authors:** N Naiyanet, S Oonsiri, C Lertbutsayanukul, S Suriyapee

**Affiliations:** 1 Department of Radiology, Faculty of Medicine, Chulalongkorn University, Bangkok, Thailand; 2 Department of Radiology, King Chulalongkorn Memorial Hospital, Bangkok, Thailand

**Keywords:** Electronic portal imaging, IMRT, CTV-to-PTV margin, head and neck cancer

## Abstract

**Purpose:**

To measure the interfraction setup variation of patient undergoing intensity-modulated radiation therapy (IMRT) of head and neck cancer. The data was used to define adequate treatment CTV-to-PTV margin.

**Materials and methods:**

During March to September 2006, data was collected from 9 head and neck cancer patients treated with dynamic IMRT using 6 MV X-ray beam from Varian Clinac 23EX. Weekly portal images of setup fields which were anterior-posterior and lateral portal images were acquired for each patient with an amorphous silicon EPID, Varian aS500. These images were matched with the reference image from Varian Acuity simulator using the Varis vision software (Version 7.3.10). Six anatomical landmarks were selected for comparison. The displacement of portal image from the reference image was recorded in X (Left-Right, L-R), Y (Superior-Inferior, S-I) direction for anterior field and Z (Anterior-Posterior, A-P), Y (S-I) direction for lateral field. The systematic and random error for individual and population were calculated. Then the population-based margins were obtained.

**Results:**

A total of 135 images (27 simulation images and 108 portal images) and 405 match points was evaluated. The systematic error ranged from 0 to 7.5 mm and the random error ranged from 0.3 to 4.8 mm for all directions. The population-based margin ranged from 2.3 to 4.5 mm (L-R), 3.5 to 4.9 mm (S-I) for anterior field and 3.4 to 4.7 mm (A-P), 2.6 to 3.7 mm (S-I) for the lateral field. These margins were comparable to the margin that was prescribed at the King Chulalongkorn Memorial Hospital (5-10 mm) for head and neck cancer.

**Conclusion:**

The population-based margin is less than 5 mm, thus the margin provides sufficient coverage for all of the patients.

## INTRODUCTION

Radiotherapy for head and neck cancer requires accuracy of radiation dose to the target volume. Setup reproducibility in the head and neck area is particularly important due to the proximity of many critical organs. The introduction of new technology such as intensity modulated radiation therapy (IMRT) and 3-D conformal radiation therapy poses new challenges for delivering intended target dose while minimising dose and toxicity to critical normal structures. This is accomplished by conforming the treatment fields to the target volume, using appropriate margins to account for treatment uncertainties. To determine these margins between the clinical target volume (CTV) and field borders, the concept of the planning target volume (PTV) has been introduced by the International Commission on Radiation Units and Measurement (ICRU) [1]. The PTV is the CTV plus a margin to allow for geometrical uncertainty in its shape and variations in its location relative to the radiation beams due to organ mobility, organ deformation and patient setup variations. The common methods to monitor treatment accuracy are visual comparison of simulation film or DRRs (prescription) and port film (treated) or electronic portal imaging. However, the image quality of DRR images is not good enough to set as reference image due to large slice thickness (5mm). We elect to use simulation image by using conventional simulation to verify setup isocenter before moving the patient to the treatment room. Megavoltage film measurements are rather time consuming and not always very accurate. Significant improvements in both accuracy and efficiency of detecting and correcting setup errors can, in principle, be achieved by using electronic portal imaging devices where the setup is verified prior to each treatment and, in some situations, also during the treatment. Since 2005, EPIDs have become available in the Division of Radiation Oncology at King Chulalongkorn Memorial Hospital, so the portal imaging from EPID was used to check the setup accuracy in this study.

At present, a CTV-to-PTV margin ranging from 5 mm to 10 mm is prescribed to patients undergoing IMRT of head and neck cancer at our division. However, a too small CTV-to-PTV margin will result in a geometrical miss at some or even all treatment fractions. It, therefore, becomes increasingly important to define adequate CTV-to-PTV margin. RTOG protocol H-0022 [2], suggests using a uniform CTV-to-PTV margin of at least 5 mm until the institution-specific uncertainty has been evaluated. Therefore, the purpose of this study is to measure interfraction setup variation in head and neck cancer patients undergoing IMRT. The data will be used to define adequate CTV-to-PTV margin.

## MATERIALS AND METHODS

This study was performed on 9 head and neck cancer patients, treated with dynamic IMRT, 6 MV X-ray beam from Varian Clinac 23EX of 120 leaves MLC at King Chulalongkorn Memorial Hospital from March 1^st^ to November 30^th^, 2006. Treatment fields encompass primary tumour as well as lymph nodes at risk. All the patients were immobilised with a TYPE-S^TM^ thermoplastic mask covering head, neck and shoulders, which was fixed to the treatment couch. Prior to treatment, all patients had three images of setup field, which were two orthogonal, anterior-posterior (AP) and lateral image at the upper neck level, and the other AP field at the shoulder level. The simulation images were acquired on the Acuity digital simulator and transferred into VARiS Vision as the reference images. Weekly portal images of three setup fields were acquired for each patient with amorphous silicon EPID. All portal images were matched with the reference images using the VARiS Vision (version 7.3.10) software.

### Portal image analysis by anatomical matching

Before collecting the patient data, the quality control of image software had been performed to verify the accuracy of the software, using perspex (PMMA) phantom attached with the marker. The images were collected in anterior and lateral directions for both simulator and EPID. Then the program of Anatomy Matching was used to verify the accuracy of the program by looking at the deviation of the marker. The matching showed good agreement with the deviation within 0.5 mm.

**Figure 1:**
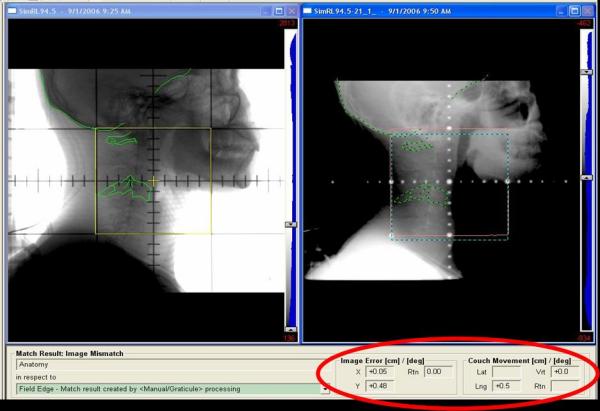
(Left) Simulator image of a right lateral setup field with contours outlined skull bones, C1 and C4. (Right) Corresponding treatment portal image matched to skull bones. An additional match was performed on this image to C1 and C4.

**Figure 2:**
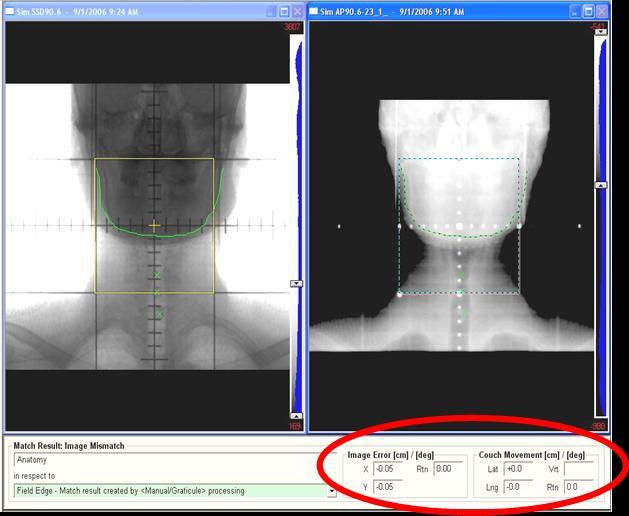
(Left) Simulator image of an anterior setup field with contour outlined mandible and spinous process. (Right) Corresponding treatment portal image matched to mandible. An additional match was performed on this image to spinous process.

**Figure 3:**
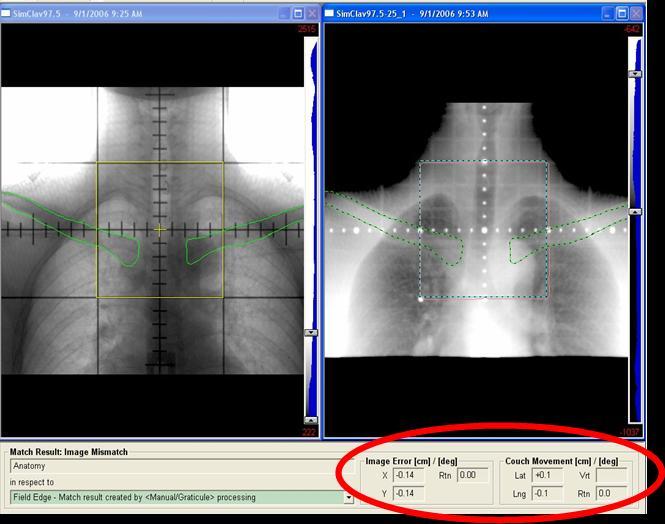
(Left) Simulator image of an anterior setup field at shoulder level with contour outlined clavicle. (Right) Corresponding treatment portal image matched to clavicle.

Comparison between a simulator image set as reference image and a portal image was done using Anatomy Matching. Anatomy Matching is used to find a small patch of image around each point in the reference that matches an identical patch in the portal image. In this study, we created an anatomy layer that was required for the matching process. Anatomical contours of bony landmarks, which were skull bones, the first cervical vertebral body (C1) and the fourth cervical vertebral body (C4) for lateral field and mandible, clavicle and spinous process for anterior field, were drawn manually on each reference image. Then the system aligned the portal images and the reference image anatomically according to the defined match points on the matching anatomy layer. An anatomy match object is produced and superimposed on the portal image and subsequently shifted until a satisfactory match is achieved. The patient misalignment is now visible and indicated in the Image Mismatch panel (Figure 1-3).

#### Setup error for head-and-neck patients

Displacements of isocenter in X (Left-Right, L-R) and in Y (Superior-Inferior, S-I) directions were measured on anterior portal images, whereas, in Z and Y direction were measured on lateral portal images. After the anatomical matching was performed on the treatment fields for an individual patient, mismatch data were recorded into a Microsoft^®^ Excel spreadsheet.

The reported X, Y and Z displacement of isocenter between simulation and treatment was decomposed into the appropriate shifts along each body axis. In this study, positive shifts correspond to shifts inferior, left and anterior.

#### Systematic error and random error for individual patient and population

For each individual patient, measurement of the displacement between simulator image and one single treatment session represents the total variation in patient positioning for the treatment session considered. This displacement is a combination of both the systematic and the random error.

The systematic error represents displacement that was persistent during the whole treatment course. For an individual patient, the systematic error (∑) was calculated as the average displacement of a particular reference structure and direction between simulation and treatment during the whole treatment course,

(1) $$Systematic\text{​}\;Error=\sum ind=\frac{\sum_{i}^{N}\Delta i}{N}$$

where N represents the total number of portal images acquired for a particular field and Δ *_i_* is the calculated displacement for the *I* th treatment fraction.

The random error represents day-to-day variations during the treatment course. For each individual patient, the random error (σ) was calculated as the dispersion around the systematic error,

(2) $$Random\;\;\;Error=\sigma ind=\sqrt{\frac{\sum_{i}^{N}{\left(\Delta i-\sum \right)}^{2}}{\left(N-1\right)}}$$

The systematic and random errors for each patient were calculated, using Eqs. (1) and (2) [4]. For the whole population, the population systematic errors (Σ_pop_) for a particular isocenter and dire action were expressed by the standard deviation (SD) from the values of the average displacement of all individual patients (Σ_ind_). While the population random error was expressed by the SD from all individual random error (σ_ind_) [5].

### Margin calculation

According to ICRU report 62 [1], the CTV-to-PTV margin should account for internal motion and variations in the size, shape and position of the CTV (internal margin) and setup uncertainties (setup margin) in the patient’s position relative to the beam. For this study, it was assumed that the location of the PTV is adequately represented by bony structures, due to the anatomy in the head and neck region, thus, the internal target motion is considered negligible. Population-based margins were calculated for all patients based on the equations of van Herk [6]. To ensure a minimum dose of 95% to the CTV for 90% of the patients, a one-dimensional margin of 1.64∑_pop_ + 0.7σ_pop_ is suggested, where ∑_pop_ and σ_pop_ are defined by Gilbeau [5]. The calculated CTV-to-PTV margins were then compared to a value 5-10 mm based on traditional margins used in the King Chulalongkorn Memorial Hospital.

## RESULTS

A total of 135 images (27 simulation images and 108 portal images) and 405 anatomical matches was evaluated. Table 1 (a)-(c) and Table 2 (d)-(f) represent sample spreadsheets used to calculate deviations along the L-R, S-I and A-P axes for each patient. The systematic (Σ_ind_) and random (σ_ind_) calculated using Eqs. (1) and (2) are also listed in Table 1 and 2. The individual systematic error (Σ_ind_) ranged from -3.5 to 2.9 mm, -2.8 to -4.5 mm and -7.4 to 2.5 mm along L-R, S-I and A-P direction, respectively. The individual random error (σ_ind_) ranged from 0.4 to 4.8 mm, 0.4 to 3.8 mm and 0.2 to 3.1 mm along the L-R, S-I and A-P axes, respectively (data not shown). The population-based margin ranged from 2.4 to 4.5 mm (L-R), 3.4 to 4.9 mm (S-I) for anterior field and 3.4 to 4.7 mm (A-P), 2.6 to 3.7 mm (S-I) for the lateral field. The summary of the population-based statistics (Σ_pop_ and σ_pop_) and one-dimensional population-based margins are presented in Table 3 and Table 4

**Table 1:** Example spreadsheets for a right lateral field matched over the course of six fractions to skull bones (a), C1 (b) and C4 (c), respectively. ΔAP and ΔSI represent the deviations in the A-P and S-I direction of each anatomical landmark between simulation images and portal images. Positive shifts correspond to anterior and inferior shifts while negative shifts correspond to posterior and superior shifts. (a) Skull bone (b) C1 (c) C4

**Date**	**Fraction**	**ΔAP (mm)**	**ΔSI (mm)**
3/1/2006	1	-3	2.3
3/3/2006	2	-2.7	0.3
3/7/2006	3	-2.4	2.9
3/14/2006	4	-2.9	1.9
3/21/2006	5	-2.9	1.9
3/28/2006	6	-2.4	2.4
	Σind	-2.7	2.0
	σind	0.3	0.9

**Date**	**Fraction**	**ΔAP (mm)**	**ΔSI (mm)**
3/1/2006	1	-3.5	1.4
3/3/2006	2	-3.7	0.3
3/7/2006	3	-4.8	2.4
3/14/2006	4	-2.4	2.4
3/21/2006	5	-3.9	1.4
3/28/2006	6	-2.9	1.4
	Σind	-3.5	1.6
	σind	0.8	0.8

**Date**	**Fraction**	**ΔAP(mm)**	**ΔSI (mm)**
3/1/2006	1	-4.9	1.8
3/3/2006	2	-3.7	1.8
3/7/2006	3	-4.8	3.4
3/14/2006	4	-2.4	2.4
3/21/2006	5	-3.9	1.4
3/28/2006	6	-2.9	2.4
	Σind	-3.8	2.2
	σind	1.0	0.7

**Table 2:** Example spreadsheets for an anterior field matched over the course of six fractions to mandible (a), clavicle (b) and spinous process (c), respectively. ΔLR and ΔSI represent the deviations in the L-R and S-I direction of each anatomical landmark between simulation images and portal images. Positive shifts correspond to left shifts and negative shifts correspond to right shifts. (a) Mandible (b) Clavicle (c) Spinous process

**Date**	**Fraction**	**ΔLR(mm)**	**ΔSI (mm)**
3/1/2006	1	-0.5	2.9
3/3/2006	2	1.4	1
3/7/2006	3	1.9	0.5
3/14/2006	4	1	-2.9
3/21/2006	5	0	-2.9
3/28/2006	6	0.5	-1
	Σind	0.7	-0.4
	σind	0.9	2.3

**Date**	**Fraction**	**ΔLR(mm)**	**ΔSI (mm)**
3/1/2006	1	-1.9	1
3/3/2006	2	0.5	0.5
3/7/2006	3	3.9	1.4
3/14/2006	4	-2.9	-1.4
3/21/2006	5	-3.4	-1.9
3/28/2006	6	-3.9	-1.9
	Σind	-1.3	-0.4
	σind	3.0	1.5

**Date**	**Fraction**	**ΔLR(mm)**	**ΔSI (mm)**
3/1/2006	1	-2.4	3.9
3/3/2006	2	0.5	3.4
3/7/2006	3	0	3.9
3/14/2006	4	-3.4	-2.4
3/21/2006	5	-4.8	-1
3/28/2006	6	-4.8	-1
	Σind	-2.5	1.1
	σind	2.3	2.9

**Table 3:** Population-based statistics (Σ_pop_ and σ_pop_) and one-dimensional population-based margins (1.64Σ_pop_+ 0.7σ_pop_) calculated for each anatomical structure of all patients along the A-P and S-I axes for lateral field.

**Lateral Field**	**Skull bone**		**C1**		**C4**	
	ΔAP (mm)	ΔSI (mm)	ΔAP (mm)	ΔSI (mm)	ΔAP (mm)	ΔSI (mm)
Σ_pop_	1.64	1.74	2.29	1.06	2.07	1.20
σ_pop_	0.99	1.20	1.36	1.24	1.73	1.61
Margins	3.4	3.7	4.7	2.6	4.6	3.1

**Table 4:** Population-based statistics (Σpop and σpop) and one-dimensional population-based margins (1.64Σ_pop_ + 0.7σ_pop_) calculated for each anatomical structure of all patients along the L-R and S-I axes for anterior field.

**Anterior Field**	**Mandible**		**Clavicle**		**Spinous process**	
	ΔLR(mm)	ΔSI (mm)	ΔLR (mm)	ΔSI (mm)	ΔLR (mm)	ΔSI (mm)
Σ_pop_	0.91	1.83	1.68	2.16	1.99	1.38
σ_pop_	1.24	1.51	2.27	1.94	1.78	1.55
Margins	2.4	4.1	4.4	4.9	4.5	3.4

These CTV-to-PTV margins for head and neck cancer were less than traditional 5 mm margin used in King Chulalongkorn Memorial Hospital.

## DISCUSSION

The primary objective of the present study was to measure interfraction setup variation in head and neck cancer patients undergoing IMRT using an EPID. Displacements of portal images from simulator images, set as reference images, were measured for calculating both systematic and random errors. Systematic error can arise from various factors, the most important being transfer errors from simulator to the treatment unit. Random errors are related to any accidental error during setup, due to mispositioning of the patient in the mask, movements of the patient or organ motion in the period between positioning and start of irradiation or during irradiation. Prisciandaro *et al*. [4] reported systematic errors ranging from -0.3 to -0.2 mm, -0.2 to 1.1 mm and -0.4 to 1.2 mm and random errors ranging from 3.0 to 3.6 mm, 2.2 to 3.3 mm and 2.6 to 2.7 mm, along the L–R, S–I and A–P axes, respectively, using TYPE-S^TM^ head/neck shoulder immobilisation systems. While our study has shown the systematic errors (systematic error ranging from -3.5 to 2.9 mm, -2.8 to -4.5 mm and -7.4 to 2.5 mm and for random errors ranging from 0.4 to 4.8 mm, 0.4 to 3.8 mm and 0.2 to 3.1 mm along the L-R, S-I and A-P axes) that exceed those in previous work, the random errors are comparable. The impact of systematic errors is much larger than the impact of random errors. Large systematic errors lead to a large underdosage for some of the patients while large random errors lead to a moderate underdosage for a large number of patients.

Based on the results presented in Table 3 and Table 4, the difference in one-dimensional population-based margins along S-I axis between anterior (3.4 to 4.9 mm) field and lateral (2.6 to 3.7 mm) field were observed because the clavicles, chosen for anterior field at the shoulder level were less stable than anatomical landmarks chosen for lateral field (i.e. skull bone, C1 and C4).

The secondary objective of the present study was to define adequate CTV-to-PTV margin for IMRT of head and neck cancer in our department. Ideally, the CTV-to-PTV margin should be determined solely by the magnitudes of the uncertainties involved. In practice, the clinician usually also considers abutting healthy tissues when deciding on the size of the CTV-to-PTV margin [7].

Stroom *et al*. [8] developed a different method for calculating CTV-to-PTV margin for prostate, cervix and lung cancer cases, which ensures at least 95% dose to 99% of the CTV. It appears to be equal to about 2∑ + 0.7σ for all three cases, based on the assumption that the CTV should be adequately irradiated with a high probability. In clinical practice, one might prefer a tighter CTV-to-PTV margin near a dose-limiting structure.

In this study, population-based margins were calculated for all patients based on the equations of van Herk [6] that suggested a one-dimensional margin of 1.64∑_pop_ + 0.7σ_pop_ to ensure a minimum dose of 95% to the CTV for 90% of the patients. In the present study, population-based margins ranging from 2.4 to 4.9 mm were demonstrated. As compared to our traditional margin of 5 mm in our department, it seems that we can further decrease the CTV-to-PTV margin to spare more organs at risk in the future.

## CONCLUSION

In this study, the population-based margin was less than 5 mm, thus the margin provides sufficient coverage for all of the patients. The result of this study suggests that the determination of setup variation is important for the assessment of population-based margin calculation to define adequate CTV-to-PTV margin of head and neck cancer patients and improve the confidence in patient-specific margin.
